# The effect of perceptual history on the interpretation of causality

**DOI:** 10.1167/jov.22.11.13

**Published:** 2022-10-21

**Authors:** Michele Deodato, David Melcher

**Affiliations:** 1 Psychology Program, Division of Science, New York University Abu Dhabi, United Arab Emirates; 2 Psychology Program, Division of Science, New York University Abu Dhabi, United Arab Emirates

**Keywords:** causality, serial dependence, perceptual history

## Abstract

The ability to interpret spatiotemporal contingencies in terms of causal relationships plays a key role in human understanding of the external world. Indeed, the detection of such simple properties enables us to attribute causal attributes to interactions between objects. Here, we investigated the degree to which this perception of causality depends on recent experience, as has been found for other low-level properties of visual stimuli. Participants were shown launching sequences of colliding circles with varying collision lags and were asked to report their impression of causality. We found short-term attractive and long-term repulsive and attractive effects of perceptual history on the interpretation of causality. Stimuli directly following a causal impression were more likely to be judged as causal and vice versa. However, prior judgments on less recent (>5) trials biased current perception with both positive/attractive and negative/repulsive influences. We interpret these results in terms of two potential mechanisms: adaptive temporal binding windows and updating of internal representations of causality. Overall, these results demonstrate the important role of prior experience even for causality, a fundamental building block of how we understand our world.

## Introduction

The ability to interpret spatiotemporal contingencies in perception in terms of causal relationships plays a key role in human understanding of the external world. Indeed, the detection of such simple properties enables us to reliably predict the consequences of our own and others’ actions and, more generally, attribute causality to interactions between objects (Caggiano, Fleischer, Pomper, Giese, & Thier, 2016; Scholl & Tremoulet, 2000). There is a long-standing debate over the nature of causality. The philosopher David Hume argued that causality is not directly perceived (like color or shape) but reflects a conscious inference (Hume, 1739/1896), while Immanuel Kant argued that we must already bring the a priori concept of causality to bear on perception (Kant, 1783/2004). In contrast, Michotte and others have argued that we directly perceive causality and that it depends on relatively low-level computations (Michotte, 1946/1963).

A paradigmatic example of causal inference is the “launching effect” (Michotte, 1946/1963). This involves a visual sequence in which one object (A) moves toward another stationary object (B) until they collide, at which Point A stops and B starts moving in the same direction. Strikingly, this basic visual percept elicits immediate and irresistible impressions of causality that are usually associated with higher-level cognition (Scholl & Tremoulet, 2000). In a pioneering work on visual causality, Michotte introduced the launching effect to argue that causality can be perceived (rather than inferred) as a form of gestalt-like perceptual organization, and he characterized the spatiotemporal parameters that elicit this impression (Michotte, 1946/1963; Wagemans, van Lier, & Scholl, 2006).

Michotte's research generated great interest in the factors that determine our percept of causality. Judgments of causality are strongly determined by both spatial factors, including the pathway and overlap of the items (Straube & Chatterjee, 2010; Woods, Lehet, & Chatterjee, 2012), and temporal factors, such as whether the second object starts moving at the same moment that the first item stops moving (Young & Sutherland, 2009). Both spatial and temporal overlap do not need to be exact for the impression of causality, reflecting spatial and temporal binding windows around the threshold whose extent varies independently between participants (Straube & Chatterjee, 2010). A number of studies have investigated the necessary perceptual conditions that elicit causality impressions (Buehner & Humphreys, 2010; Choi & Scholl, 2004, 2006; Young & Sutherland, 2009) and the distinction between perceived and inferred causality (Meyerhoff & Scholl, 2018; Roser, Fugelsang, Dunbar, Corballis, & Gazzaniga, 2005; Schlottmann & Shanks, 1992). In addition, other studies have focused on the neural correlates of visual causality (Blos, Chatterjee, Kircher, & Straube, 2012; Fugelsang, Roser, Corballis, Gazzaniga, & Dunbar, 2005; Roser, Fugelsang, Handy, Dunbar, & Gazzaniga, 2009; Straube & Chatterjee, 2010).

An additional question is the degree to which our interpretation of causality depends on context and recent experience. Gruber and colleagues (Gruber, Fink, & Damm, 1957), for example, asked participants to judge the collapse of a model bridge and measured the temporal threshold of causality by introducing a variable delay between the collapse of the bridge and the removal of one of its vertical supports. They reported an “anchoring” effect in the threshold of causality relative to a prior block of trials with long versus short delays (Gruber et al., 1957). Similarly, Powesland (1959) replicated this finding using launching stimuli: Long-delay practice trials tended to increase the temporal threshold of causality while no-delay practice trials had a shortening effect. Additionally, the influence of practice trials increased as their number was augmented (Powesland, 1959). These results suggest that the temporal binding window of causality is adaptive, growing or shrinking based on recent experience.

More recently, Rolfs, Dambacher, and Cavanagh (2013) employed a spatiotemporal manipulation of launching events that involved different levels of overlap at the collision between the launcher and the launched object. They found that after an adaptation period to direct (nonoverlapping) launching events, subsequently viewed events were judged more often as noncausal. Importantly, given that this negative aftereffect was restricted to the retinotopic coordinates of the adaptation stimuli, the authors argued that it may represent a canonical form of visual adaptation for the perception of causality (Johnston, 2013; Kominsky & Scholl, 2020; Rolfs et al., 2013). Such studies investigated the influence of prolonged exposure to a spatiotemporal sequence on subsequent interpretations of causality. Interestingly, there has been a growing interest in the more general principle, across many stimuli and paradigms, that perceptual judgments of a visual stimulus can be biased toward the stimulus that was presented in the previous trial (Fischer & Whitney, 2014; Liberman, Fischer, & Whitney, 2014). This positive aftereffect has been labeled “serial dependence,” and it has been suggested to act in contrast to uncertainty and environmental noise, increasing the impression of continuity in perceptual experience (Cicchini, Mikellidou, & Burr, 2018; Fritsche, Spaak, & de Lange, 2020; Samaha, Switzky, & Postle, 2019).

Here, we investigated whether causality judgments also showed serial dependence and, if so, what the temporal parameters were of this effect. To this end, we asked participants to report their impression of causality to launching sequences with a variable delay at the moment of collision. It has previously been shown that when the spatial or temporal offset is near a threshold, causality judgments vary from trial to trial (Straube & Chatterjee, 2010). Given the intrinsically perceptual nature of visual causality, we reasoned that this percept could be affected by previous experience in a manner consistent with a serial dependence effect.

## Methods

### Participants

A group of 18 individuals (10 male) participated in the experiment. This sample size was chosen based on previous studies (e.g., Roseboom, 2019). Inclusion criteria were age between 18 and 35 years (*M* = 22.5, *SD* = 4.3) and normal or corrected-to-normal vision. All participants signed an informed consent form and received either course credit or a small gift certificate for their participation. Data were collected in accordance with the Declaration of Helsinki, and the study protocol was approved by the local ethics committee (New York University Abu Dhabi Institutional Review Board).

### Experimental design

**Figure 1.**
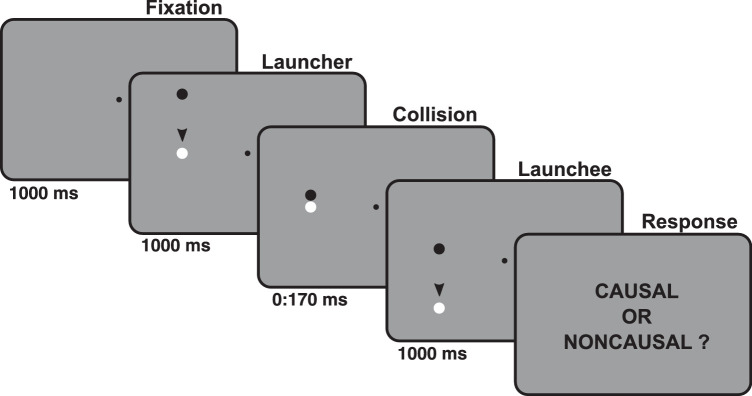
Experimental design, the launching sequence. Each trial started with a fixation; after 1 s, two circles appeared on one side of the fixation. The black circle moved for 1 s toward the white circle and then collided with it. The collision display remained frozen for a time interval that ranged between 0 and 170 ms in steps of 17 ms. After the collision, the white circle moved away for 1 s before disappearing, and the participants were presented with a question (“Causal or Noncausal?”), prompting them to report their impressions of causality through a forced-choice causal versus noncausal response.

All stimuli were presented on a 60-Hz monitor at a viewing distance of 50 cm and displayed against a uniform gray background. Each trial started with a fixation at the center of the screen, and then a launching sequence was presented. The sequence consisted of a white circle moving toward and “colliding with” a black circle, “causing” it to move in the same direction. The stimuli moved vertically either toward the bottom or toward the top on the screen and were always presented in the left visual hemifield with the starting position of the black circle aligned to the fixation (see Figure 1).

We manipulated the duration of a temporal lag at the time of collision to induce causal versus noncausal impressions. Specifically, we introduced 11 equally spaced collision lags ranging from 0 to 170 ms. Each combination of a sequence direction and collision lag was repeated 20 times, for a total of 440 randomized trials. The total time taken by the participants to complete the experiment was ∼ 30 min.

Participants were instructed to look at the fixation point while paying attention to the lateralized stimuli and then to report with a key press whether they perceived the interaction between the moving circles as causal or not. Emphasis was put on not considering this assignment as a lag detection task, but rather to focus on the impression of causality elicited by the collision. All participants were naive to the purpose of the experiment. The experimental design was implemented in MATLAB using the psychophysics toolbox (Brainard, 1997).

### Data analysis

First, for each participant, we fitted a psychometric function to the percentage of noncausal judgments with respect to the collision lags. The 50% perceptual threshold of this curve was taken as the point of subjective causality (PSC). Participants with a goodness of fit inferior to 90% were excluded from further analyses. The data from 2 participants were rejected on the basis of poor goodness of fit, leaving 16 participants for further analyses.

Second, all trials were divided in two bins based on whether they were following a causal or noncausal response, and we obtained a PSC estimate for each bin and participant. To better characterize the temporal dynamics of serial dependence, this step was then repeated considering responses at a position further backward in the trial history. Specifically, we explored this effect up to 15 trials back. This number was chosen, despite many studies focusing only on the previous trial or up to around 5 trials backward, because of recent reports of long-lasting effects of serial dependence (Manassi & Whitney, 2022) and studies showing an attractive to repulsive dependency reversal when considering less recent perceptual history (Suárez-Pinilla, Seth, & Roseboom, 2018).

The difference between the PSC of the causal and noncausal groups represents a measure of the influence of previous trials on current causality judgments. We reasoned that if recent perceptual history has no effect on current causality judgments, then the grouping/binning step would be statistically equivalent to resampling and lead to an average PSC difference of zero. On the contrary, a positive difference could be interpreted as an attractive effect (Fischer & Whitney, 2014), while a negative difference would be akin to repulsive aftereffects (Kominsky & Scholl, 2020; Rolfs et al., 2013).

### Statistical analysis

Statistical significance of the PSC difference was obtained by means of nonparametric permutation testing. Following our reasoning (see above), on each permutation (*N* permutations = 10,000), the trial order was shuffled and the PSC difference was estimated after the response-based sampling repeated, leaving us with a zero-centered normal distribution of PSC differences under the null hypothesis that perceptual history is not a significant factor in the interpretation of causality. The original PSC difference values were then compared to the null hypothesis distributions to obtain *p*-values, and false discovery rate was applied to correct for multiple comparisons.

## Results

**Figure 2.**
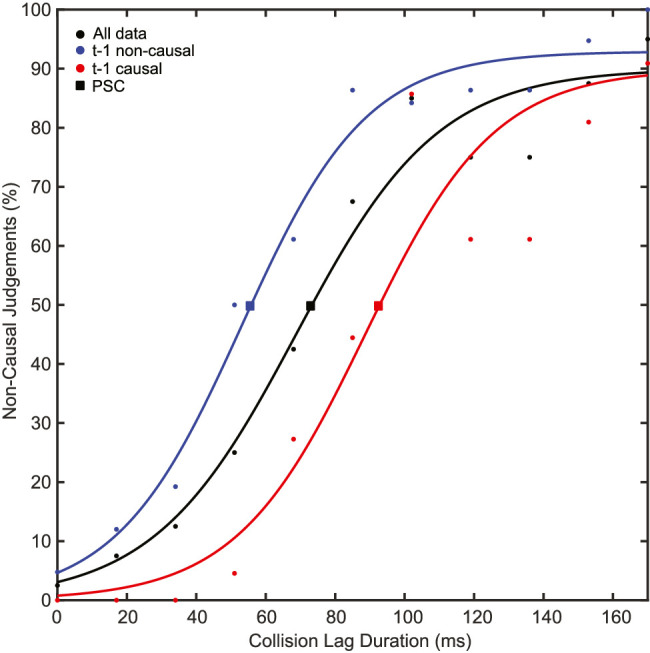
Distribution of causality judgements with respect to different collision lags for an example participant. The figure shows the psychometric function fitted to all trials (black line and dots), trials following a causal response (red line and dots), and trials following a noncausal response (blue line and dots). Squares indicate the points of subjective causality (50% threshold) of each function.

As expected, the proportion of noncausal judgments increased as a function of the collision delay duration (Figure 2). This allowed us to determine each participant's PSC by fitting a psychometric curve, yielding a mean PSC of 79 ms (*SD* = 13.05 ms). This distribution yielded roughly 50% of causal and noncausal responses (*M* = 51.2%, *SD* = 6.7%) for each participant, ensuring a similar amount of data points to reliably estimate the PSC for the two response-based data groups.

**Figure 3.**
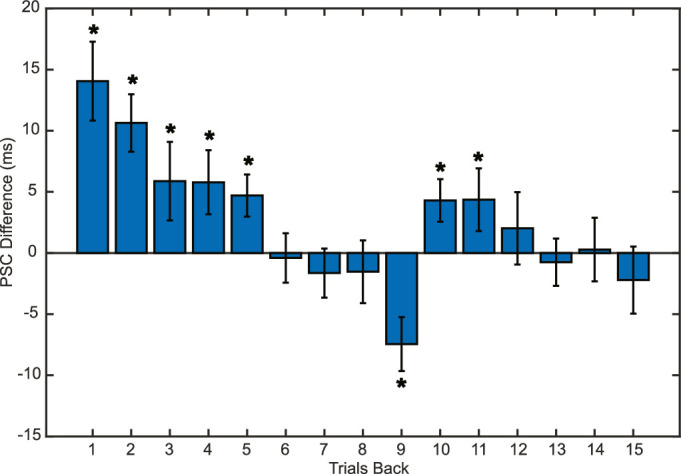
Influence of previous trial on visual causality. Error bars represent the standard error. Asterisks indicate a significant *p* value after false discovery rate correction.

Consistent with the hypothesis of serial dependency, we found a strong attractive effect in the difference between the PSC of trials directly following causal versus noncausal responses. One way to look at this result is that causal judgments may have prompted an increased “tolerance” or underestimation of the perceived lag duration in the launching sequence, thus favoring causal interpretations even for longer lag sequences. Similarly, noncausal judgments may have led to the opposite effect: an overestimation of the lag duration that results in more noncausal impressions. In other words, we found that stimuli following a causal impression were more likely to be judged as causal and vice versa. This effect was strongest in, but not limited to, the previous trial (PSC difference = 14.05 ms, *p* < 0.001; see Figure 3). When considering the backward temporal evolution of this dependency effect, the PSC difference gradually decreased but remained significant up to five trials back. Interestingly, trials in less recent perceptual history were characterized by a reversal in the direction of this difference that became significant at 9 trials back and abruptly returned to an attractive effect before tapering to a statistical zero after the 11th trial (see Figure 3).

In order to minimize the possibility of biases due to uneven distribution of trials between the collision lags in the groups, we repeated the analysis by collapsing together all the trials of all participants. This approach, which removed the risk of small bin sizes for specific participants, yielded a similar pattern in the data, with a strong attraction effect for five trials backward followed by a repulsion effect and a return to attraction*.* Given that the duration of a trial was at least 3 s (see Figure 1), this suggests that serial dependence acts for up to 15 s in our data set, in agreement with recent reports of long-lasting attractive influences of visual history on perception (Manassi & Whitney, 2022).

## Discussion

The purpose of this study was to complement findings of the effects of previous experience on visual causality by showing that even just one trial can affect our impressions of causality in terms of the collision between two objects. We replicated the finding that impressions of causality elicited by launching stimuli can be altered by introducing temporal gaps at the time of collision. More important, we found that such impressions are influenced by previous causality judgments, following a complex backward temporal dynamic. There was a large effect of the previous trial on perceptual judgments of causality. Trials immediately preceding the current stimulus showed an attractive effect, while trials in less recent perceptual history showed an abrupt change pattern similar to what was reported by Fritsche and colleagues (2020) and suggests an interaction between both attractive and repulsive biases.

The repulsive/attractive reversal suggests the involvement of different dependencies, potentially reflecting different neural mechanisms. Attractive and repulsive influences of perceptual history are usually associated with serial dependence and adaptation, respectively, and have been historically investigated as separate processes. For example, it has been shown in some paradigms that adaptation acts in retinotopic coordinates while serial dependence does not (Mikellidou, Cicchini, & Burr, 2021). Also, it has been suggested that attractive and repulsive effects occupy different temporal scales (Fritsche et al., 2020). However, recently there has been a growing number of reports showing both positive and negative biases in the same set paradigm (Chopin & Mamassian, 2012; Fritsche et al., 2020; Rafiei, Chetverikov, Hansmann-Roth, & Kristjánsson, 2021; Suárez-Pinilla et al., 2018). It has been proposed that they are caused by opposite decisional and perceptual influences (Fritsche, Mostert, & de Lange, 2017) that are due to efficient strategies for decoding and encoding of visual information (Fritsche et al., 2020), which may be caused by the motor and perceptual components of the responses (Zhang & Alais, 2020), or they could be the result of a single mechanism (Maus, Chaney, Liberman, & Whitney, 2013).

Overall, a number of reports have argued that the influence of perceptual history on current percepts reflects the need for a balance between the needs for sensitivity to novelty and taking advantage of, and reinforcing, stability in the visual environment. Given the temporal nature of the manipulation in our task, these strategies may justify the existence of adaptive temporal windows that bind together causes and consequences and could serve as a flexible mechanism for the detection of causal contingencies. A similar mechanism has been shown to operate rhythmically for the temporal integration of separate events (e.g., Benedetto, Burr, & Morrone, 2018; Ronconi, Oosterhof, Bonmassar, & Melcher, 2017).

On the one hand, given the importance and ubiquity of causal events in our everyday life, there is a strong prior probability for causal interpretation. However, we also must be sensitive to changes in temporal contiguity in order to notice when the situation changes since things do not always work the same way as expected. In this sense, perhaps both Hume and Kant were partially correct: We bring an a priori tendency to perceive events in terms of causality if they meet certain spatiotemporal parameters (à la Kant), but this perception also depends on previous experience and involves a type of inference (*d'après* Hume).

This binding of causes and consequences has been shown to affect the perception of low-level temporal and spatial properties of collision events. Specifically, temporal lags and spatial gaps are perceived as shorter than they actually are when they are embedded in a causal collision (Buehner & Humphreys, 2009, 2010). Moreover, the ability to flexibly adapt temporal binding windows has been reported for self-initiated actions and their consequences, in terms of “intentional binding” (Melcher, Kumar, & Srinivasan, 2020; Stetson, Cui, Montague, & Eagleman, 2006), as well as for multisensory integration of audiovisual stimuli (Fujisaki, Shimojo, Kashino, & Nishida, 2004). Repulsive and attractive effects have been found in the perception of timing and synchrony using different paradigms (Roseboom, 2019). This is particularly relevant considering that the effect of serial dependence on causal judgments of launching sequences cannot be easily disentangled from a history-biased estimate of synchrony or lag duration. Notably, Woods and colleagues reported that the temporal threshold for causality depends on the temporal context provided in the experiment (i.e., the temporal range of the collision lags) (Woods et al., 2012). Likewise, an effect of “causal capture” in which the launching impression can be generalized to stimuli with abnormal spatiotemporal collisions has been identified with respect to the spatial context in which launching stimuli are embedded (Scholl & Nakayama, 2002). Evidence for the existence of temporal binding windows in causality includes the finding that new information can influence our experience of the immediate past, as reflected by postdictive processes in causal perception (Choi & Scholl, 2006; Newman, Choi, Wynn, & Scholl, 2008). Such flexibility in perceiving causal relationships may have adaptive significance and justify our findings of dependencies in perceptual history.

The dynamic nature of the detection of causal events could also depend on more top-down mechanisms. For example, in the “reordering effect,” a launched object is perceived to move after the collision with a launcher, even when it starts several milliseconds before. This has been interpreted as the influence of previous knowledge and causality expectations on the perception of low-level temporal properties of causal displays (Bechlivanidis & Lagnado, 2016; Fornaciai & di Luca, 2020; Pedro, 2020). Another way to consider the detection of causality and its temporal dependencies, beyond low-level features, is as the result of matching stimulus features to internal templates of causal mechanisms (White, 2005). This view, also known as the schema-matching hypothesis, posits that the brain holds and continuously update schematic representations of object interactions based on previous experience (Roser et al., 2009; White, 2005). In the context of the launching effect, everyday experience with colliding objects should create a template of collisions as contiguous temporal lags and coherent spatial trajectories. Indeed, Roser and colleagues found that implausible launching sequences generate a greater neural response associated with the update and maintenance of an online mental model of the recent past with respect to plausible collisions (Roser et al., 2009). This constant update of an internal template of collision dynamics would generate variability in the impressions of causality and context-dependent changes in its temporal threshold, therefore offering another plausible explanation for our finding of serial dependence in several launching presentations with heterogeneous temporal features.

In summary, we demonstrate that causality judgments for a classic Michotte launching display show serial dependency. Both positive and negative effects were found, depending on the number of previous trials considered. We propose that repulsive and attractive effects of perceptual history on the interpretation of causality could arise from two independent sources or from their combined activity: on the one hand, the low-level consequences of flexible temporal binding windows and, on the other hand, the top-down maintenance and updating of internal representations of causality.

## Conclusions

Visual causality is affected by perceptual history in a more complex way than previously reported. We found a combination of adaptive and repulsive dependencies, and even just a launching sequence can greatly affect future impressions of causality. Generally, our findings add to a growing literature speaking for the complex interaction of attractive and repulsive influences of perceptual history on perception. We propose that these effects in visual causality could be the result of the online calibration of flexible binding windows, of the update mechanism for a template of causal collisions or of their interaction.
